# Electroporation of cDNA/Morpholinos to targeted areas of embryonic CNS in *Xenopus*

**DOI:** 10.1186/1471-213X-7-107

**Published:** 2007-09-27

**Authors:** Julien Falk, Jovana Drinjakovic, Kin Mei Leung, Asha Dwivedy, Aoife G Regan, Michael Piper, Christine E Holt

**Affiliations:** 1 Department of Physiology, Development and Neuroscience, University of Cambridge, Downing Street, Cambridge CB2 3DY, UK; 2 The Queensland Brain Institute, The University of Queensland, St Lucia, QLD, 4072, Australia

## Abstract

**Background:**

Blastomere injection of mRNA or antisense oligonucleotides has proven effective in analyzing early gene function in *Xenopus*. However, functional analysis of genes involved in neuronal differentiation and axon pathfinding by this method is often hampered by earlier function of these genes during development. Therefore, fine spatio-temporal control of over-expression or knock-down approaches is required to specifically address the role of a given gene in these processes.

**Results:**

We describe here an electroporation procedure that can be used with high efficiency and low toxicity for targeting DNA and antisense morpholino oligonucleotides (MOs) into spatially restricted regions of the *Xenopus *CNS at a critical time-window of development (22–50 hour post-fertilization) when axonal tracts are first forming. The approach relies on the design of "electroporation chambers" that enable reproducible positioning of fixed-spaced electrodes coupled with accurate DNA/MO injection. Simple adjustments can be made to the electroporation chamber to suit the shape of different aged embryos and to alter the size and location of the targeted region. This procedure can be used to electroporate separate regions of the CNS in the same embryo allowing separate manipulation of growing axons and their intermediate and final targets in the brain.

**Conclusion:**

Our study demonstrates that electroporation can be used as a versatile tool to investigate molecular pathways involved in axon extension during *Xenopus *embryogenesis. Electroporation enables gain or loss of function studies to be performed with easy monitoring of electroporated cells. Double-targeted transfection provides a unique opportunity to monitor axon-target interaction *in vivo*. Finally, electroporated embryos represent a valuable source of MO-loaded or DNA transfected cells for *in vitro *analysis. The technique has broad applications as it can be tailored easily to other developing organ systems and to other organisms by making simple adjustments to the electroporation chamber.

## Background

*Xenopus laevis *is a model system widely used to study vertebrate development. Much of our understanding of early embryo patterning and tissue induction has come from this model, and *Xenopus *has provided many important insights into neuronal development. However, many of the molecules involved in neuronal differentiation also play crucial roles in early development [1,2]. Therefore, the classical approach of injecting blastomeres with DNA/mRNA or antisense oligonucleotides (morpholinos, MOs) is of limited use for studying axon guidance as it interferes with gene function during early development and frequently leads to abnormal embryogenesis. In some cases, this problem can be circumvented by the use of inducible or tissue specific promoters [3-6] but selective expression during a specific time-window in selected populations of cells remains difficult and levels of expression often decrease with time due to plasmid dilution during cell division [7,8]. Ideally, to test the function of a specific molecule in axon guidance, its function should be disrupted exclusively during the period of axonogenesis. To this end, lipofection has proven useful to introduce DNA in the developing eye and brain of stage 19–24 *Xenopus *embryos [8,9] and viral infection using vaccina virus has also been used in stage 40–48 *Xenopus *embryos [10,11]. However, each of these techniques has drawbacks, such as the low efficiency of transfection of lipofection and the low expression level and reproducibility of vaccinia viral infection [12]. Electroporation does not suffer from these limitations. Indeed, its ease of use combined with efficient and accurate spatio-temporal targeting quickly established electroporation as superior to most other methods of genetic manipulations in chick embryos [13-16].

In addition to DNA and RNA, electroporation can be used to deliver dsRNA, RNAi, antisense morpholinos (MO), dyes and proteins [17-21]. This large repertoire and the ability to introduce several types of molecules at the same time have provided new paradigms for monitoring gene expression, cell morphology, movements and lineage, as well as efficient means for interfering with protein and microRNA function [13,19,22-25]. As a result, chick electroporation has made major contributions to the understanding of gene regulation, cell proliferation, migration and differentiation, and more generally of the underlying mechanism of nervous system patterning and neuronal wiring [13,22,26-28]. Electroporation methods have now been adapted for use in many animal models including mouse [13,29], rat [29], zebrafish [30,31], ascidian [32], *hydra *[33] and *drosophila *[34]. In *Xenopus*, electroporation has been successfully used to introduce DNA into the brains of late tadpole embryos (stages 44–48) [10,12,35] and RNA into the CNS of early neurula embryos (stage 12.5) [36,37]. Although a previous study reports that stage 25–29/30 embryos can be successfully electropermeablized [38], electroporation has only been used during this developmental window to enhance lipofection [39]. Thus, no electroporation protocol has been described for the intermediate developmental ages (stages 21–40) that span the critical 40 h window of brain wiring, when most of the major axon tracts are formed in the *Xenopus *CNS [40-44].

We describe here a detailed electroporation procedure to introduce efficiently both DNA and MOs to restricted regions of the brain and eye between stages 21 and 35/36. This protocol relies on the design of "electroporation chambers", tailored to individual embryonic stages, which allows reproducible and efficient large or targeted electroporation of different regions of the CNS. We demonstrate that projection neurons and their targets, both intermediate and final, can be selectively manipulated by multiple targeted electroporations or a combination of electroporation and lipofection. As such, electroporation can be a reliable and efficient tool to examine gene function during CNS differentiation. Finally, we provide evidence of the potential benefits of electroporation for the study of axonogenesis *in vitro*.

## Results and discussion

### Electroporation chambers enable reproducible and efficient electroporation

**Figure 1:**
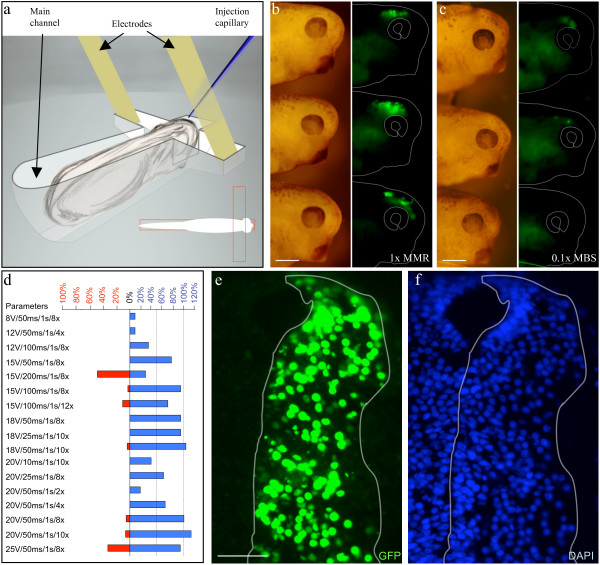
**Efficient DNA transfection of stage 26–28 *Xenopus *embryos**. a: Schematic representation of the experimental setup. Embryos were placed in the main channel of the electroporation chamber, while the electrode tips (0.5 mm wide) were positioned in the transverse channel. A diagram of the setup is presented as an insert with channel (outlines in red). b, c: Representative images of embryos electroporated in 1× MMR and 0.1× MBS. Bright field images (left panel) and GFP fluorescence (right panel) of living embryos 12 h after electroporation. No morphological abnormalities are observed. d: Histograms presenting the relative transfection efficiencies (blue) evaluated from observation of embryos as shown in c and d. The percentage of embryos showing macroscopic damage (red) was recorded for each condition. Different parameters are listed in the following order: Voltage, pulse duration, interpulse space and number of pulses. e, f: Electroporation resulted in a high percentage of transfected cells without affecting brain microanatomy. Nls-GFP signal (e) was observed in many nuclei (f) from the ventricle to the most superficial layer 48 h after electroporation. The transfected hemi-brain was outlined in white. Scale bars: 400 μm in b and c; 100 μm in e.

Efficient and reproducible electroporation relies primarily on the precision of the injection of DNA. To control injection accuracy, stage 21–35/36 embryos must be held in the desired position and submerged in a drop of medium, as they easily deform and are highly sensitive to drying. Previously published electroporation procedures could not be used because they do not permit accurate orientation of the embryos [38] nor take into account the soft-tissue vulnerability or morphology of the targeted stages [12,36]. Therefore, we developed electroporation chambers tailored individually to the size and morphology of embryos from stages 21 to 35/36. The basic design of the chambers consists of two channels carved perpendicular to one another in Sylgard in the shape of a cross (Figure 1a). The embryo is held in the longitudinal channel while the electrodes are placed in the transverse channel. The size and geometry of the longitudinal channel was optimized for each embryonic stage to provide a "snug fit" for the embryo and full immersion in medium. The position of the transverse channel insures reproducible placement of the electrodes along the anterior-posterior axis of the embryo and its depth controls the amount of electrode surface in contact with the medium, and thus the dorso-ventral extent of the embryo exposed to the electric field. The length of the transverse channel is designed so that when electrodes are placed at each end, electroporation efficiency is maximized while damage to the embryo is minimized. In addition, the spacing and immobilization of the electrodes in the transverse channel enable accurate positioning prior to injection. This allows the electric field to be applied immediately after DNA injection which is critical for minimizing diffusion and backflow of the injected solution through the opening made by the capillary [19,45,46].

### Electroporation leads to efficient transfection in *Xenopus*

To determine the optimal conditions for *Xenopus *electroporation, voltage, frequency and duration of electrical pulses were systematically varied using the experimental set-up illustrated in Figure 1a (embryos were kept at 18°C). 93% of the embryos (n = 34) injected at stage 26–28 into the third ventricle with a solution containing 1 μg/μl of green fluorescent protein (GFP) encoding plasmids exhibited bright GFP expression 12 h after being exposed to 8 square-pulses of 20 V 50 ms applied every second (20 V/50 ms/1 s/8 x) (Figure 1b). Efficient transfection required a high conductivity electroporation medium as the success rate dropped 2.5-fold (n = 26) when 0.1× **M**odified **B**arth's **S**aline (MBS) was used instead of 1× MBS or 1× **M**odified **M**odified **R**inger's (MMR) (Figure 1b and 1c). The high conductance of the medium surrounding the low conducting embryo could enhance electroporation by preventing the decrease of electric field inside the embryo as shown on cellular spheroids [47]. As summarized in Figure 1d (blue), a series of 4 or more 15–20 V pulses with a duration of 25–100 ms each led to >60% electroporation success rate. Electroporation efficiency consistently increased in proportion to pulse number, voltage and duration (when below 200 ms). A decrease in voltage or pulse duration could be partially compensated for by increasing the number of pulses.

**Figure 2:**
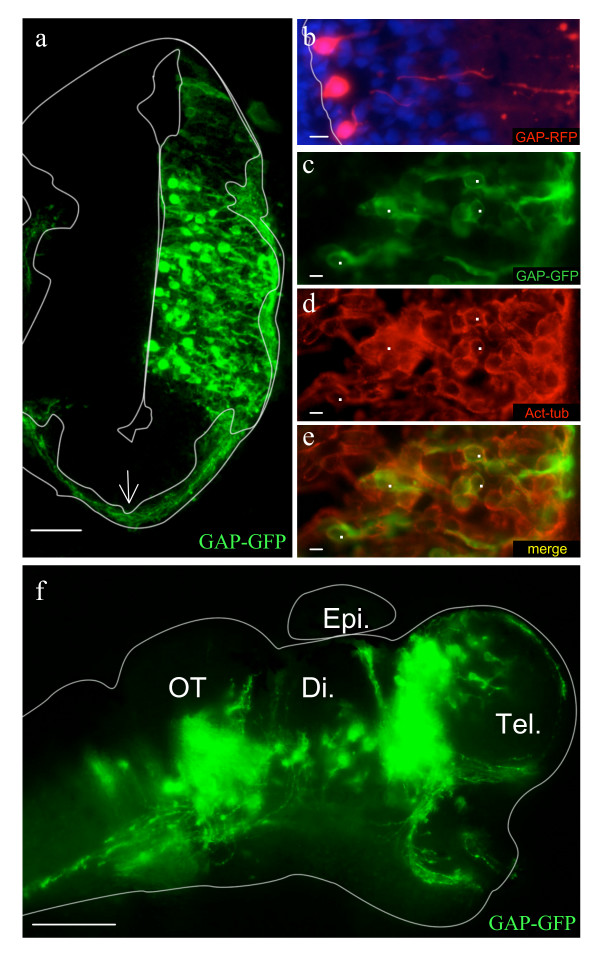
**Cell types and morphology of the transfected cells**. a: Membrane-tethered GFP (GAP-GFP) delineated the processes of transfected neurons including the axons (the ventricle and neuropil are outlined in white). The arrow indicates a bundle of axons travelling in the neuropil). b: Radial-glia like morphology of GAP-RFP transfected cells lining the ventricle. c-e: Co-expression of GAP-GFP (c) and acetylated-tubulin (d) in superficial layers (e- merge). f: Wholemount brain preparation from an electroporated embryo showing different axon tracts. The brain outline was drawn based on the corresponding bright field image. Di., diencephalon; OT, optic tectum; Tel., telencephalon; Epi., epiphysis. Scale bars: 100 μm in f; 50 μm in a; 10 μm in b-e.

To further characterize the efficiency of electroporation, nucleus-targeted GFP (nls-GFP) was transfected to quantify the fraction of GFP-expressing versus non-expressing cells on transverse brain sections counterstained with a nuclear stain (DAPI). 48 h after electroporation (20 V/50 ms/1 s/8 x), the average fraction of cells expressing GFP per section was 47.1 ± 2.5% in the transfected region (n = 47 sections 6 embryos; Figure 1e and 1f). Transfected cells were scattered along 50–70% of the dorso-ventral axis and throughout the whole neuroepithelium. At this stage, the brain comprises a proliferative region adjacent to the ventricle lumen (ventricular zone) surrounded by layers of migrating and differentiating neurons (mantle zone). 48 h post-electroporation, transfected cells were present in both regions. GFP-expressing cells residing in the superficial third of the brain, populated by differentiated neurons, represented 31.8 ± 1.5% of total labeled cells (n = 32). However, the fraction of transfected cells in the superficial half of the brain increased with pulse duration (see additional file 1 a & b). To check the morphology of transfected cells and further characterize their cell types, we transfected membrane-targeted GFP or RFP (GAP-GFP and -RFP). As expected, the GFP signal was found from the ventricle to the neuropil (Figure 2a). Cells lining the ventricle could be seen extending radial process towards the pia, typical of dividing cells (Figure 2b). Transfected neurons appeared to differentiate normally as they expressed the neuronal marker acetylated tubulin, and sent long processes into the neuropil (Figure 2c–e). Furthermore, several axon tracts could be recognized in a whole-mount view of the brain (Figure 2f).

Finally, we assayed the potential adverse side effects of electroporation. Electroporation did not increase either the embryo death rate or the occurrence of morphological abnormalities, provided the pulse voltage remained under 25 V and the pulse duration under 100 ms (Figure 1d red). The anatomy of the embryos and their brains appeared normal on transverse sections at all time points after electroporation tested (Figure 1f). Some pyknotic nuclei were observed in highly transfected embryos in the first 24 h post-electroporation. Therefore, TUNEL staining was used to assess cell death on sections. 24 h after exposure to 20 V/50 ms/1 s/8 x and 18 V/25 ms/1 s/10 x, the average number of TUNEL positive cells per section was 5.21 ± 0.35 (n = 48) and 2.8 ± 0.48 (n = 28) respectively (see additional file 1g). This compares favorably with an average of 3.5 ± 0.27 cells/section (n = 63) in control embryos and indicates that the electric pulses are relatively harmless *per se*. However, other parameters such as DNA purity, embryo quality, manipulation and injection are critical for minimizing cell death. Thus, electroporation of ventricular injected DNA led to efficient transfection of both the dividing ventricular region and differentiated neurons without increasing cell death or affecting their morphological differentiation.

### Electroporation at different stages produces rapid and long-lasting transgene expression

Neuronal differentiation and initial establishment of the major axonal projections progress rapidly from stage 20 to 40 and, during this period, the neuroepithelium undergoes major reorganization with post-mitotic neurons migrating away from the ventricular surface to the superficial layers. To achieve fine temporal resolution, the electroporation procedure should be similarly efficient across different time-windows. Therefore, we compared the efficiency of the electroporation protocol over different stages.

**Figure 3:**
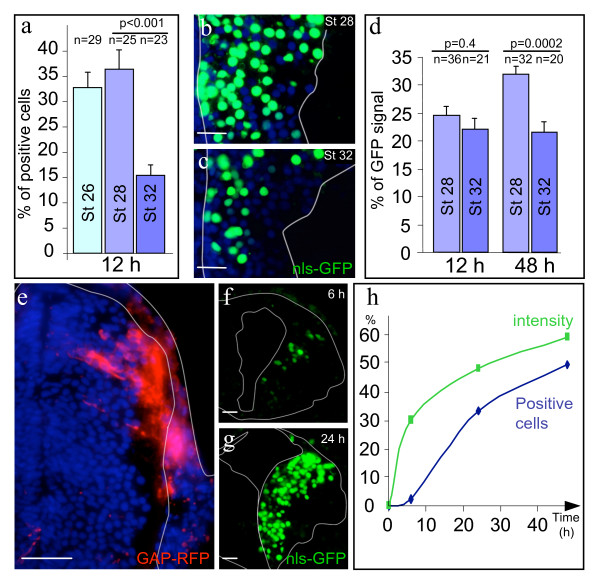
**Electroporation of stage 21–35/36 embryos leads to rapid expression of transgenes**. a: Electroporation efficiency decreased with increasing embryonic stage. Percentages of nls-GFP positive cells 12 h after transfection at stage 26, 28 or 32 (n represents the number of sections analyzed from 3 embryos). Similar results were obtained at 48 h post electroporation (data not shown). b-d: Distribution of transfected cells depended on the stage of embryos electroporated. Distribution of nls-GFP transfected cells 48 h afterwards in embryos electroporated at stage 28 (b) and 32 (c). Note that the density of cells (DAPI) is lower laterally. d: Histograms showing decreases in the fraction of cells transfected in the superficial third of the brain when embryos were electroporated at stage 32 as compared to stage 28. e: A cluster of superficially located cells can be selectively transfected by injecting the DNA solution under the skin (the pia and epidermis are outlined in white). f-h: Time course of GFP expression in embryos electroporated at stage 29/30 (20 V/25 ms/1 s/8 x). The fractions as well as mean intensities of GFP positive cells were quantified (h) from sections (examples: f and g) (15 sections from 3 embryos were analyzed for the 6 h and 48 h time points and 39 sections from 3 embryos for the 24 h time-point). Differences between the time points were statistically significant using a Mann-Whitney test; probabilities are indicated together with the standard error (S.E.M). Outlines of the brains are presented (ventricle on the left). Scale bars: 100 μm in e; 50 μm in b, c, f and g.

Embryos were electroporated following intraventricular pCS2GFP-DNA injection at stages ranging from 21 to 35/36 in chambers specially adapted to their morphology. External inspection of embryos under the fluorescent strereomicroscope showed that the fraction of embryos exhibiting bright GFP expression 12 h after electroporation was >70%, regardless of the stage at which the electroporation was performed. Despite these similar levels at a gross level, analysis of the GFP positive cell fraction (nls-GFP) on transverse sections revealed that a sharp decrease in efficiency occurs at stage 32 (Figure 3a). In addition, the GFP-expressing cells from late (stage 32) electroporations were distributed unevenly in the neuroepithelium. First, there was a marked decline in the number of transfected cells in the ventral brain. Indeed, the dorsal shift of the GFP-expression center of mass (relative to the DAPI nuclear marker) significantly increased 1.5-fold between stages 28 and 32. Secondly, 48 h after electroporation, fewer GFP-positive cells can be found in the superficial region of the neuroepithelium closest to the pia (Figure 3b–d). Several factors may contribute to the observed changes. As the brain develops, the ventricle lumen expands and post-mitotic cells migrate away from the ventricular surface to differentiate in superficial layers. Consequently, many cells are distant to the injection site, making them less likely to be transfected. In addition, a larger ventricle means a lower intraventricular concentration of injected DNA, which will restrict the transfection to cells lining the ventricle and decrease the electroporation efficiency overall. In agreement with this, doubling the injection volume enhances the electroporation success rate by 1.25 (assessed as in figure 1b; n = 12). However, if the local DNA concentration was the only factor involved, a stage-dependent distribution of GFP positive cells would be expected shortly after electroporation. In fact, 12 h after electroporation the fraction of GFP positive cells located in the superficial half of the brain was similar in embryos electroporated at stage 28 and 32 (24.4 ± 1.6, n = 36 and 21.9 ± 2.1, n = 20 sections respectively). The delayed onset of the stage-dependent difference in deep-superficial distribution suggests that it results at least partly from developmental changes in patterns of cell proliferation and migration that occur after electroporation.

In order to gain access to the cells situated close to the pia, we delivered DNA to the pial surface by injecting under the skin epidermis instead of intraventricularly (stage 29/30 embryos). Subcutaneous injections, followed immediately by electroporation, efficiently and selectively transfected cells in superficial layers of the brain (Figure 3e).

Overall, GFP expression in embryos electroporated between stages 21 to 35/36 displayed similar kinetics. In whole embryos, the GFP signal can first be detected 5–6 h after electroporation. This signal progressively intensifies and spreads over the subsequent 36 h, and remains high for several days. Nuclear GFP was used to quantify the GFP expression at different time points on transverse sections of embryos electroporated at stage 29/30. A progressive increase in both the fraction and the average intensity of the GFP positive cells was observed between 6 and 48 h post-electroporation (Figure 3f–h). The sigmoid shape of GFP kinetics likely reflects the requirement for a progressive accumulation of the GFP signal in the transfected cells to reach the detection threshold, combined with proliferation of transfected progenitors. Interestingly, 6 h after intraventricular injection/electroporation (20 V/50 ms/1 s/8 x) of stage 29/30 embryos, 36.7 ± 3.2% of the GFP positive cells were found in the superficial half of the brain, suggesting that electroporation efficiently targets both proliferating and differentiated cells. The wide range of stages amenable to electroporation, combined with the quick onset of transgene expression, demonstrates that this technique provides precise temporal control.

### Controlling spatial targeting to study axon guidance: the retino-tectal projection

The spatial selectivity allowed by electroporation has proven useful for axon guidance studies [17,23,48-51]. Thus, using the well-characterized retinotectal projection system, we next asked if our procedure could enable selective transfection of retinal ganglion cells (RGCs) and/or the regions through which their axons travel. Normally, axons from RGCs exit the eye, travel along the optic nerve to enter the brain at the ventral diencephalon. After crossing the midline at the optic chiasm, they extend dorsally through the optic tract in the diencephalon before turning caudally to reach the optic tectum, where they arborize and form synapses.

**Figure 4:**
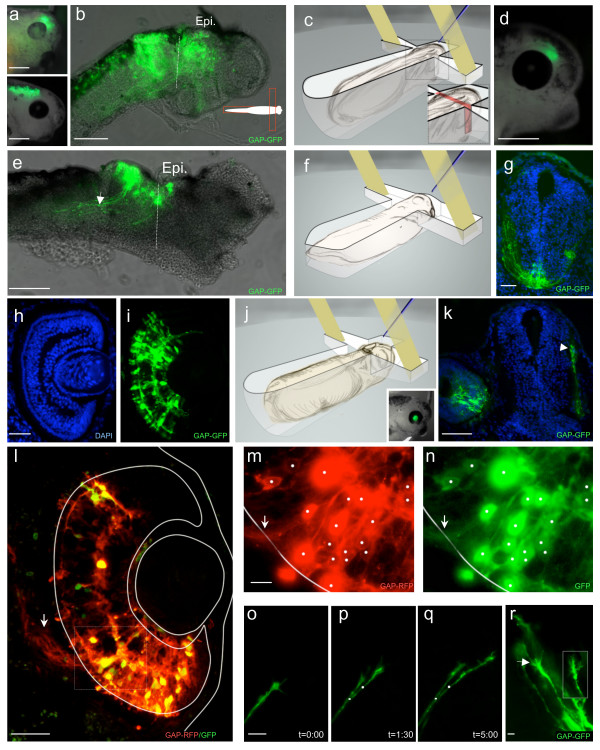
**Using electroporation to study retino-tectal projections *in vivo***: a-b: Regions of the brain can be differentially targeted by sliding the embryo in the main channel (compare upper and lower panels in a). When the caudal part of the head was exposed, most of the optic pathway was electroporated (b). c-e: The transfected area can be restricted by reducing the amount of embryo area directly facing the electrodes. The modified chamber used to restrict electroporation is depicted in c (note the narrowing of the transverse channel in the inset), and a representative example of GFP expression 12 h post electroporation in a live embryo is shown in d. GFP expression in the tectum is shown on a wholemount dissected brain (e). Axons emanating from these neurons can be clearly observed (arrow). The dashed line delineates the diencephalon/mesencephalon boundary. The transfected area is restricted to the OT (dorsal mesencephalon). f-g: Electrodes can be placed dorsal and ventral to the embryo to target the ventral or dorsal part of the brain. A frontal section through the midbrain (g) demonstrating that ventral populations can be targeted by placing the embryo on its side in the specifically designed chamber represented in f. h-r: Retinas can be electroporated without affecting eye development. 48 h post electroporation, GAP-GFP was detected in all the retinal layers and outlined different retinal cell types with their characteristic morphologies (h-i). Eye microanatomy appeared normal (h). Eye-targeted electroporation can be performed by placing the embryo ventral side up, so that the eye but not the brain faces the electrodes (j). Eye-specific electroporation can be performed with limited brain transfection. Insert: side view of a transfected embryo 24 h after eye-targeted electroporation. GFP signal was detected in the eye and the RGC axons navigating to the tectum (arrow) but not in the brain on frontal sections (k). l-n: Co-electroporation of pCS2GAP-RFP with pEGFP. Most of the GAP-RFP positive cells (m) are also EGFP positive (n). Double positive cells are marked with white dots and the arrows point to axons leaving the retina. Outlines of the retina and lens were drawn from the corresponding DAPI counterstainings. After GAP-GFP electroporation, axons can be monitored using time-lapse microscopy (o-q) and growth cone morphology can be analyzed (r) in wholemount brain preparations. Axons were monitored as they entered the tectum. Initial positions of the two growth cones are indicated (white dot and rectangle). Time is in hours. Epi., epiphysis. Scale bars: 400 μm in a, d and insert j; 200 μm in b and e; 100 μm in k; 50 μm in h, i and l; 25 μm in o-q; 10 μm in m and n; 5 μm in r.

Since only the region lying between the two electrodes is efficiently electroporated, different areas can be selectively electroporated by sliding the embryo forward or backward in the main channel to expose the rostral or caudal part of the head (Figure 4a and 4b). This configuration gives rise to large transfected areas, extending rostro-caudally over 568 ± 40.5 μm (n = 13). Taking advantage of the insulating property of Sylgard, the electroporated region can be restricted by narrowing the transverse channel (158 ± 17.6 × 89 ± 8.7 μm, n = 14), making specific electroporation of the embryonic tectum or diencephalon feasible (Figure 4c–e). In addition, the relative orientation of embryos to the electrodes can be changed to drive DNA towards different regions. Using modified chambers, the ventral-most regions of the brain, which are usually difficult to electroporate, can be targeted, allowing electroporation of the optic chiasm region (Figure 4f and 4g).

To specifically electroporate the eye, embryos were placed belly up so that the eye, but not the brain, was aligned with the electrodes (Figure 4j). This avoided non-targeted electroporation of the brain, which is important as the lumen of the eye vesicle, where the DNA injection is made, communicates directly with the brain ventricles at early stages (Figure 4j insert and k). Eye specific electroporation was successfully performed over a range of stages from 22 to 35/36 without affecting eye development (Figure 4k, see additional file 1ee and 1ff [electroporated at stage 24, 28 and 32 respectively]). The success rate, evaluated 12 h after electroporation, was over 80%. 48 h after electroporation, all layers of the retina were transfected and cellular morphology within the retina appeared normal (Figure 4h and 4i). Interestingly, when eyes were electroporated at stage 32, GFP-positive cells were widely distributed in early stage 33/34 retina only 6 h after transfection (see additional file 1cc and 1dd). Eye-targeted electroporation yielded high levels of transgene co-expression when pCS2GFP and pCS2GAP-RFP plasmids were injected in a 1:1 ratio. 95.4 ± 1.2% of the GFP positive cells were RFP positive and 81.8 ± 3.1% of the RFP expressing cells were also GFP positive (n = 313 and n = 366 cells respectively from 10 sections). More importantly, the co-electroporation efficiency remained high even if different types of plasmids were mixed (Figure 4l–n). Co-electroporation enables multiple perturbations as well as easy monitoring of the transfected cells. Indeed, RGC axons can be easily analyzed both in transverse sections (Figure 4k) and in the whole brain (Figure 4o) after GAP-GFP electroporation. In addition, GAP-GFP transfection enables powerful time-lapse analysis of extending retinal axons and growth cone dynamics to be performed *in vivo *(Figure 4o–r) [52]. Fixed sample analysis as well as live monitoring of GAP-GFP expressing RGC axons show that electroporation does not perturb axonal growth, navigation or branching. Thus, electroporation is suitable for manipulating and monitoring RGC axons at stages when lipofection has proven to be difficult.

**Figure 5:**
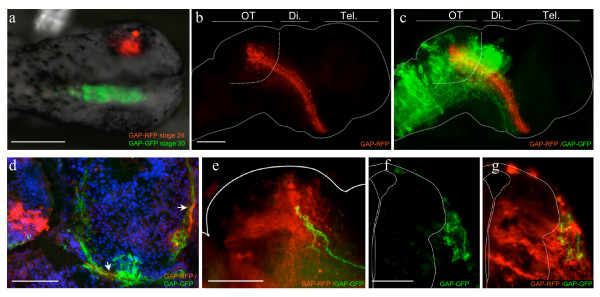
**Both retinal projection neurons and their substrate pathway can be manipulated separately in the same embryo**. a-d: Eye-targeted electroporation can be combined with brain electroporation. a: A dorsal view of an embryo doubly transfected. Retinal axons (red in b and c) navigate normally to the tectum, passing through a transfected region of the diencephalon (green in c) (dashed line indicates the OT boundary). Eye- and ventral-targeted electroporation can be combined (d). Frontal section showing axons from the transfected retina (red) that have crossed the transfected midline (GFP-transfected) and growing dorsally towards tectum (arrow). e-g: Electroporation can be performed on embryos lipofected in the eyes. e: High magnification of two GFP lipofected axons passing through a cluster of electroporated tectal cells. f and g: Frontal sections of an embryo lipofected in the eye and electroporated in the brain. Retinal axons in the dorsal brain (green: f, g) traversed the transfected cells (red: g). Outlines of brains in wholemounts (b, c, e) and sections (f, g) were drawn based on bright field images and DAPI counterstainings respectively. Epi., epiphysis; Di., diencephalon; OT, optic tectum; Tel, telencephalon. Scale bars: 400 μm in a; 100 μm in b-g.

Finally, we asked if both the eye and the pathway where retinal axons grow (e.g. optic tract, optic tectum) could be manipulated separately within the same embryo. We electroporated eyes at stage 24 with GAP-RFP and brains 8 h later at stage 30 with GAP-GFP. We found that dual-electroporation produced specific expression both within the eye and pathway. Importantly, dual electroporation did not decrease embryo viability or cause abnormal development (n = 21), and did not affect brain anatomy (see additional file 2ee). Furthermore, eye-specific electroporation can be combined with either large or area-specific brain electroporation (Figure 5a–d). Similarly, brain electroporation was successfully performed on eye-lipofected embryos (Figure 5e–g). Thus, dual-electroporation provides a way to separately control transgene expression in the retinal axons versus the substrate pathway enabling *in vivo *analysis of axon-target and axon-pathway interactions.

### Targeted loading of antisense morpholinos by electroporation

In addition to DNA transfection, intracellular delivery of antisense morpholinos (MOs) was tested. MOs are an effective tool to knock-down protein expression in *Xenopus *through blastomere injection [53,54] but their inability to be taken up through the plasma membrane has limited their use at late stages. Standard MOs are uncharged and, therefore, cannot be electroporated. However, MOs can be fluorescently tagged for visualization and, fortuitously, the tag introduces a charge making them amenable to electroporation [19]. Electroporation of MOs in chick has been used to study nervous system development, and single cell electroporation in the brain of late-stage *Xenopus *embryos has been reported [12,19].

**Figure 6:**
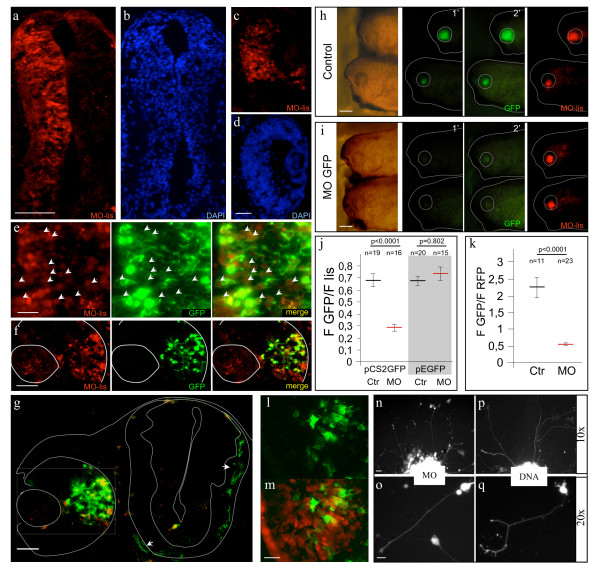
**Introducing Morpholinos into young *Xenopus *tadpoles by electroporation and *in vitro *approaches**. a-d: Frontal sections of embryos 24 h after electroporation with lissamine-tagged MO. Large numbers of cells can be loaded with MO in both the brain (a) and the eye (c). Microanatomy of both structures appears normal (b and d). e-f: Co-electroporation of pCS2GAP-GFP with lissamine-tagged special delivery MO. e: A higher magnification image of a co-electroporated brain. The MO signal was de-saturated in Photshop in order to facilitate observation of MO and membrane GFP co-expression (arrowhead). f: An image of eye-targeted co-electroporation illustrating the extent of co-electroporation and the sizes of MO and DNA electroporated regions. g: Frontal section of a MO/GFP co-electroporated embryo showing that GFP can be used to trace the axons of electroporated cells (arrowheads indicate axons at different points in their pathway). h and i: Examples of embryos electroporated with pCS2GFP in the presence (i) or absence (h) of anti-GFP MO. Morphology of the eye appeared normal in both conditions (left panel). The GFP signal was sharply reduced in the anti-GFP MO condition when analyzed 12 h after electroporation (central panels). A decrease in electroporation efficiency was not a confounding factor in this experiment as the Special Delivery lissamine-tagged MO control is efficiently loaded in both conditions (far right panel). j: Quantification of results presented in h and i (n indicates the number of embryos analyzed). Anti-GFP MO only affects expression of pCS2GFP but not of pEGFP (Clontech). k: Anti-GFP MO was co-electroporated with GFP and GAP-RFP. 48 h after electroporation, GFP and RFP fluorescence was quantified on sections and the ratio between the two calculated. (n refers to the numbers of sections quantified [3 embryos were analyzed for control and 6 for MO]). Statistical analysis: Mann-Whitney test; probabilities are indicated together with the S.E.M. l-m: Sections through an eye lipofected with GFP (green, l and m) and subsequently loaded with lissamine-tagged MOs (red) using electroporation (merge, m). n-q: Electroporated embryos can be a source of modified cells for *in vitro *studies. Explants and cells cultured from MO (n and o) or DNA (GFP) (p and q) electroporated embryos. Scale bars: 400 μm in h; 100 μm in a; 50 μm in d, f, and g; in 25 μm e and m; 20 μm in n; 10 μm in o.

Lissamine-tagged MOs were electroporated into both the brains and the eyes of stage 22 to 33/34 embryos with a success rate of over 80% with all four settings used (20 V/50 ms/1 s/8 x, 18 V/50 ms/1 s/10 x, 18 V/25 ms/1 s/10 × or 15 V/50 ms/1 s/10 x). In transverse sections, MO-loaded cells were evenly distributed throughout the width of the electroporated side of neuroepithelium, and along most of its dorso-ventral axis (Figure 6a and 6b). With eye-targeted electroporation, MO-positive cells were found in all of the cellular layers of the retina (Figure 6c and 6d). In all conditions tested, MO fluorescence was still detectable 48 h after electroporation.

Plasmids were also successfully co-electroporated with negatively charged MOs (both fluorescein- and special delivery lissamine-tagged) (Figure 6e–g). For example, 48 h after eye-targeted electroporation (GFP 0.7 μg/μl [0.26 pmol/μl], MO 0.25 mM), 94.4 ± 1.4% of the GFP expressing cells were MO positive (n = 597 cells from 20 sections from 3 embryos). However, in this condition the MO positive domain was slightly larger than the GFP-expressing domain (Figure 6f), resulting in 65.4 ± 2.3 MO-loaded cells expressing the GFP (n = 863 cells). Finally, as GFP accumulates progressively the degree of co-localization will change slightly with time and should be taken into consideration.

At a cellular level, electroporated MOs seem to diffuse evenly around the cytosol and into the nucleus. Although short neuronal processes could be visualized with the MO fluorescence, longer processes were usually so weakly labeled that they were difficult to follow. However, co-transfection with GAP-GFP highlighted axons emanating from the electroporated RGCs, making monitoring of axons from MO-loaded cells feasible (Figure 6g).

Finally, we took advantage of the high co-electroporation efficiency to test the ability of the loaded MO to down-regulate translation of its target mRNA. A pCS2 plasmid encoding GFP was electroporated with or without a MO designed to block GFP expression [55]. Using this MO directed against the pCS2+ and cytoplasmic GFP sequences flanking the GFP start codon, we observed a 4-fold decrease of the GFP signal both in the eye and the brain when the MO against GFP was co-electroporated with pCS2GFP (n = 24, n = 30 respectively, data not shown). However, this decrease could be due to biased electroporation and/or nonspecific effects of the anti-GFP MO (such as cell death or general translation inhibition). To rule out such problems, the experiments were performed on the targetable (pCS2) and non-targetable GFP (pEGFP) in the presence of a control tagged MO to assess the electroporation efficiency. Co-electroporation of the GFP MO only decreased the expression of the targetable plasmid (Figure 6h–j). The decrease of the fluorescent ratio of the GFP over GAP-RFP signal in the GFP MO electroporated eyes further supports a specific effect of the MO on the GFP (Figure 6k). In conclusion, our electroporation procedure enables efficient loading of MOs without impairing their activity *in vivo*. This suggests that, similar to chick, controlled spatio-temporal MO knock-down approaches could be achieved by electroporation in early tadpole *Xenopus *embryos. Furthermore, electroporation allows sequential modifications of gene function when used in combination with other techniques such as lipofection (Figure 6l and 6m).

### *In vivo *electroporation provides source of transfected/MO-loaded neurons for *in vitro *studies

The embryonic *Xenopus *brain is extremely small, posing challenges for obtaining a sufficiently large number of cells to perform dissociated cell electroporation protocols [56,57] and alternative transfection methods have low efficiencies [58]. For example, MO uptake by *Xenopus *retinal cultures is inefficient even when specific transmembrane trafficking molecules, such as Endo-Porter (GeneTools) are used (data not shown). Thus, most *Xenopus *transfected or MO-loaded cells used in culture have been obtained from embryos injected at early blastomere stages [59]. However, premature death or abnormalities of injected embryos limit the spectrum of MOs or constructs that can be used to analyze later events *in vitro*. Therefore, we cultured explants or dissociated cells from different parts of brains electroporated with GAP-GFP DNA and/or fluorescently tagged control MOs (fore-, mid- or hind-brain). As shown in Figure 6n–q, both MO-loaded and DNA transfected cells can be successfully cultured and up to 40% of the cultured cells showed expression. Moreover, the positive-expressing explants and dissociated cells were readily detected, even at low magnifications suggesting that intracellular levels of the MO and the DNA were high. In culture, MOs could be readily seen in axons and growth cones (Figure 6o), and could still be detected after 2 days *in vitro*. This makes *in vivo *electroporation a potent source of transfected cells for *in vitro *approaches.

## Conclusion

We describe here an optimized procedure to electroporate different brain regions and the eye from stage 21 to 35/36 *Xenopus *embryos. Both MOs and DNA were delivered with high efficiency and with limited side effects. Electroporation enables both over-expression and knock-down studies to be performed in a spatiotemporally controlled manner. Furthermore, the high co-electroporation (DNA-DNA or DNA-MO) efficiency makes perturbation of several genes feasible and could be useful for identifying and monitoring events in the MO or DNA electroporated cells such as pathfinding or axon branching analysis. In addition, MO-DNA co-electroporation enables "rescue" experiments to be performed. Finally, using different electroporation protocols or DNA concentrations, expression levels can be kept low enough to avoid mis-localization and/or toxicity of over-expressed markers, or maximized to reach efficient concentration of dominant-negative proteins.

The electroporation chambers we designed confer several advantages. First, they enable a large number of embryos to be electroporated rapidly in a reproducible way (1–3 min per embryo). Chambers can be readily made to fit embryos of different ages, and appropriate placement of the embryo within the chamber allows different parts of the developing nervous system to be targeted. Furthermore, chambers can also be made to accommodate zebrafish embryos for which electroporation protocols have been recently developed (see additional file 2a–ca-c) [30,31]. Thus, our method has a wide range of prospective applications, both in *Xenopus *and in other organisms. Indeed, targeting of various other regions of interest for axon guidance (telencephalon, spinal cord) and double brain-targeted electroporations were successfully performed (see additional file 2d–id-i).

One main advantage of our protocol is that electroporation can be controlled spatiotemporally, which means that secondary defects arising from early gene manipulations can be avoided. Indeed, the present protocol provides the degree of targeting precision (around 150 μm^2^) required to selectively electroporate eye or brain regions in *Xenopus *embryos. As electroporation efficiency remained high at all the stages tested, the described parameters can be used to investigate gene function at a critical time for nervous system development. Electroporation also leads to quicker detectable expression of the DNA than most available techniques [9,11,12]. This rapid onset of transgene expression is particularly useful since in many cases only several hours are needed for an axon to complete its growth. As RNA can be successfully electroporated (data not shown), the delay between electroporation and protein expression could be further shortened. Finally, spatiotemporal control of expression could be further refined by using specific promoters [38].

Lastly, electroporation of previously electroporated or lipofected embryos enables sequential modification of the same region, or a combination of specific modifications of both neurons and the environment through which their axons navigate. The ability to genetically manipulate both the presynaptic neurons and the pathway/targets of their axons in the same embryo will provide a valuable new experimental paradigm for investigating axon-pathway and axon-target interactions *in vivo *and *in vitro*. For instance, co-electroporation of suitable makers in double transfected embryos may provide unique insights into the cellular interaction *in vivo *between axons and the environment, or between axon terminals and their synaptic partners.

## Methods

### Animals

Oocytes obtained from adult female *Xenopus laevis *injected with human chorionic gonadotropin hormone (Sigma) were fertilized *in vitro*. Embryos were raised in 0.1× MBS until they reached the desired stage. Stages were determined according to Nieuwkoop and Faber [60].

### Plasmids and Morpholinos

Expression plasmids pCS2GAP-GFP and RFP [61,62], pCS2GFP [8,55], pCS2nls-GFP [63], pEGFP (Clontech) were prepared from *Escherichia Coli *cultures using the Qiagen Midi DNA preparation kit (Qiagen) and resuspended in water. When concentrations above 3 μg/μl were required, the plasmid preparations were concentrated by isopropanol precipitations.

Morpholino oligonucleotide paired to a complementary carrier DNA (Special Delivery) directed against the pCS2GFP was a gift from M. Perron [55]. Crude and Special Delivery standard control (Ctr) MOs (5'CCTCTTACCTCA-GTTACAATTTATA3') fluorescently tagged with lissamine (liss) or carboxyfluorescein were purchased from GeneTools. 1 mM stock solutions were prepared and stored at -20°C. Stock solutions were heated at 65°C for 5 min prior to dilution.

### Electroporation chamber

The electroporation chambers consist of two intersecting channels carved in the shape of a "**†**" in a 0.8 cm layer of silicon elastomer coating the bottom of a 35 mm plastic petri dish (Sylgard 184, Dow Corning, USA) (see additional file 3). This material (Sylgard) was preferred over others by virtue of its mechanical resilience and electrical resistance. Sylgard is sufficiently stable to allow repetitive use of the chamber and soft enough to ensure that embryos are not damaged when placed carefully into the chamber. A total of 8 out of 34 chambers originally tested were selected. The selected chambers were successfully reproduced from negative imprints of the original ones and copies can be provided upon request (see additional file 3). The geometry of the chamber varies depending on the stage and targeting (see additional file 4a). For stage 28–30 embryos, the longitudinal channel is 7 mm long, 1 mm wide and has a maximal depth of 1 mm. The transverse channel, at the ends of which electrodes should be placed, is 4 mm long, 0.8 mm wide and 0.2–0.5 mm deep.

### Electroporation Protocol

Embryos had their vitelline membrane removed and were placed in fresh 0.1× MBS before being anaesthetized in the electroporation medium (0.4 mg/ml MS222 in 1× MBS or 1× MMR). 1× MMR: 100 mM NaCl/2 mM KCl/1 mM MgSO_4_/2 mM CaCl_2_/5 mM Hepes/1 mM EDTA. 1× MBS: 88 mM NaCl/1 mM KCl/2.4 mM NaHCO_3_/10 mM Hepes/0.8 mM MgSO_4_/0.33 mM Ca(NO_3_)_2_/0.4 mM CaCl_2_. Anaesthetized embryos were individually transferred into the transfection chamber in a drop of medium, placed into the main channel of the chamber and excess medium was gently removed. Homemade flat-ended 0.5 mm wide platinum electrodes (Sigma, 26788-1G, see additional file 4b and 4c) were placed into the transverse channel.

Pulled borosilicate glass capillaries (1 mm OD-0.78 ID, GC100TF10, Harvard Apparatus; puller Pul-1, World Precision instrument) were back-filled with MO (0.1–0.5 mM in water) and/or DNA solutions (0.5–2.5 μg/μl in water). In the case of the subcutaneous injections, higher DNA concentrations were used (3–6 μg/μl). In some cases, methylcellulose was added to limit the diffusion of the injected solution and to increase the targeting [46]. Fast Green was added to DNA but not to MO solutions as it has been shown to inhibit MO electroporation [19]. The injection capillary tip was positioned so that the targeted region lay inbetween the tip and the positive electrode.

Depending on the stage, 100–300 nl (subcutaneous), 50–100 nl (intraventricular) or 10–30 nl (eye specific) of DNA(s) and/or MO solution was injected using an air-pressured injector (Picospritzer II, Intracel). The tip of the capillary was broken with fine forceps under a stereomicroscope so that it released 5–8 nl per pulse. The capillary was removed just before the first electric pulse of the series was delivered by the square wave pulse generator (TSS20 OVODYNE electroporator, Intracel). After the pulse series was completed, the electrodes were removed and the embryo gently collected from the chamber in a large drop of electroporation medium. Electroporated embryos were then placed in sterile 0.1× MBS and grown at 18°C. To avoid damage due to handling, embryos were moved with large round-tip glass tools and were transferred in and out of the chamber with a large plastic pipette. Both the capillary and electrodes were manipulated using manual micromanipulators (Fine Science Tools).

### Brain Sections, DAPI and TUNEL staining and image processing

Embryos were fixed with 4% PFA in PBS over night at 4°C and then rinsed with PBS. Subsequently, fixed embryos were equilibrated in 15% then 30% sucrose/PBS solutions and embedded in Tissue-Tek OCT compound (Sakura). 10 μm cryostat sections were collected on Superfrost slides (VWR) and dried for 60 min prior to staining. Sections were post-fixed in ethanol/acetic acid (2/1 volumes) for 5 min at -20°C prior to TUNEL labeling. TUNEL labeling (Apoptag fluorescein kit S7110, Intergen Company) was performed according to the manufacturer's recommendations. The sections were incubated in DAPI at 1/10000 (D9542, Sigma) for 5 min in 0.1% Triton/1× PBS at room temperature (RT) and washed 3 times in PBS before mounting. Blocking was done with 1% BSA/10% Goat serum/0.1% Triton in 1× PBS for 30 min at RT. For labeling of differentiated neuronal cells, the sections were incubated with anti-acetylated tubulin (6-11B-1, Zymed, stock: 0.5 mg/ml) for 2 h at RT (1/200 in the blocking buffer) and visualized with a Cy3 anti-mouse secondary antibody (AP 124C, Chemicon) (1/1000 in the blocking buffer).

Sections were mounted in Fluorosave medium (Calbiochem), and photographed. All images were acquired from grayscale cameras (ORCA-ER, Hamamatsu) using Open *lab *software (Improvision) and processed in Photoshop (Adobe). For TUNEL quantification, all TUNEL labeling co-localizing with DAPI positive structures in the brain were counted on sections (where the eye was present). The statistical analysis was performed in InStat3 (Graphpad Software Inc).

### Evaluation of the transfection efficiency

For testing the electroporation parameters, embryos from different test conditions were injected with the same volume using the same capillary. Electroporation using the standard setting (20 V/50 ms/1 s/8 x) was always performed at the end of the test series as a control.

12 h after electroporation, the success rate was estimated on live anesthetized embryos under a fluorescence stereoscopic microscope (MZFLII, Leica). Each embryo was scored according to the fluorescence intensity and spread of the signal (0 = no signal, 0.25 = dim, 0.5 = high but restricted, 1 = high and widespread). As absolute efficiency varies with DNA preparations and embryo batches, embryos electroporated with the standard setting were scored first to set the index. Results from different experiments were normalized to the standard settings (100%). The pictures presented and archived were taken under the same conditions (same magnification, time after electroporation and exposure). Embryos exhibiting any apparent damage such as smaller eye, local head depression, defect in eye pigmentation or persistent skin peeling were scored as damaged.

The fraction of transfected cells was quantified on serial frontal sections of embryos 6 h, 12 h, 24 h or 48 h after electroporation with nls-GFP. Sections were screened at low power (5×) to identify the rostral most and caudal most positive sections. All inclusive sections were then photographed at 20× (Eclipse 80i, Nikon) using fixed acquisition parameters separately set for the 24 h (for analysis of electroporation kinetics) and 12 h (for analysis of stage and pulse parameters) time points (Orca, Hamamatsu, Open *lab*, Improvision). Regions of interest (ROIs) corresponding to the hemi-neural tube and superficial regions of the brain were outlined based on DAPI counter-staining and used for subsequent quantification. Thresholds were set for DAPI and GFP fluorescence intensity and the total area in ROIs above threshold was calculated. Thresholds for DAPI and GFP were calibrated so that the ratio of GFP area to DAPI area matched the manual percentage count at 24 h (kinetics) or 12 h (stages and pulse parameters). The centers of mass of DAPI and GFP signals were also calculated. All the quantifications were done in ImageJ (NIH).

### Morpholino knock-down of GFP expression

Embryos were injected separately with: (1) 0.7 μg pCS2GFP or 0.7 μg pCS2GFP+0.33 mM GFPMO (eye and brain); (2) 0.7 μg pCS2GFP+0.1 mM liss-CtrMO, 0.7 μg pCS2GFP+0.33 mM GFPMO+0.1 mM liss-CtrMO, 0.7 μg pEGFPC1+0.1 mM liss-CtrMO or 0.7 μg pEGFPC1+0.33 mM GFPMO+0.1 mM liss-CtrMO (eye); (3) 0.7 μg pCS2GFP+0.7 μg GAP-RFP or 0.7 μg pCS2GFP+0.7 μg GAP-RFP+0.33 mM GFPMO (eye).

12 h after electroporation, images of intact living embryos were acquired and levels of GFP expression were quantified. For the first set of experiments (1), the integral of GFP fluorescence was calculated for the eye region. For the second set (2), a circular ROI encompassing the eye was drawn from the corresponding bright-field pictures and used to determined the mean intensity level of the red (electroporation control) and green (GFP expression) channels. The green to red ratio was calculated for all embryos that exhibited a mean fluorescence intensity over background threshold in the red channel. All acquisitions and quantifications were done blind.

In the third set of experiments (3), the expression of GFP and GAP-RFP was measured on serial coronal sections 48 h after electroporation. Images were acquired and thresholds were set identically between the conditions and the GFP/GAP-RFP ratio was calculated. All quantifications were performed in ImageJ and all statistical analysis was done in InStat3 (Graphpad Software Inc).

### Electroporation of lipofected or electroporated embryos

Stage 19–20 eye primordia were lipofected as described previously with GAP-GFP plasmid mixed with DOTAP (Roche) [8]. These lipofected embryos were then electroporated with GAP-RFP at stage 28 as described above. Embryos first electroporated at stage 24 or 28 (GAP-RFP) were allowed to recover at room temperature for several hours before being electroporated at stage 29/30 or 32 (GAP-GFP).

### Time-lapse *in vivo *microscopy

Electroporation was performed on stage 28 embryos using the standard protocol. When reaching stage 39, embryos were anaesthetized and prepared for live imaging as described previously [64]. Briefly, the eye and skin covering the contralateral brain were removed to expose the transfected axons. The embryo head was placed in 0.05 mg/ml MS222/1× MBS filled chamber formed by a gene frame (ABGene, AB 0576) placed on an oxygen permeable slide (Permanox, Nalgen Nunc, 16005). Only samples with a few isolated axons were selected for subsequent live imaging. Image acquisition was performed on a Nikon Optiphot-2 microscope equipped with a 20× Plan NeoFluar objective and Orca-ER cooled CCD camera (Hammamatsu). To minimize phototoxicity, acquisitions were made with neutral density filters on and short exposure times (50–100 ms). Z-stacks were acquired every 10 min.

### Cell culture

14 h-20 h after electroporation, brains (fore-, mid-, and hind-brain) and eyes were dissected from electroporated embryos [65] and cut into 2–4 explants. In the case of dissociated primary cultures, tissues from embryos under stage 30 were dissociated in calcium free medium (0.4 mM EDTA) [66] using fire polished Pasteur pipettes. For embryos older than stage 30, their tissues were incubated for 6–8 min in trypsin solution (Gibco) before being mechanically dissociated. The trypsin was inactivated in 10 times its volume of 10% FBS medium prior to trituration. Both explants and dissociated cells were cultured in 60% L15/10% FBS/1% PSF (100 U/ml penicillin, 100 μg/ml streptomycin and 0.25 μg/ml fungizone; Gibco) on glass coverslips coated with 100 μg/ml poly-L-lysine (Sigma) and 10 μg/ml laminin (Sigma). The cultures were analyzed 24 h and 48 h after plating.

## Competing interests

The author(s) declares that there are no competing interests.

## Authors' contributions

JF designed, performed and analyzed the experiments presented except the *in vivo *time-lapse imaging which was carried out by JD. JD also participated in establishing the protocol for eye specific transfection and optimized it for later stages. K-M L has made various attempts to load MO in RGCs which led to investigate the potential of electroporation. K-M L provided all control MOs and her expertise in using MO. Both JD and K-M L independently repeated some of the experiments presented. AD, AGR and MP performed pioneer electroporation experiments and AD and MP designed the first set of electrodes. JF drafted the manuscript with inputs from all the authors. CEH contributed to the design and coordination of the study and assisted with the writing of the manuscript. All authors read and approved the final manuscript.

## Supplementary Material

Additional file 1 **Supplementary Figure 1**. Distribution of early GFP-expressing cells in the brain and eye and stage dependency of eye-targeted electroporation. Click here for file

Additional file 2 **Supplementary Figure 2**. Potential applications of electroporation to other animal models and projection systems. Click here for file

Additional file 3 **Supplementary Methods**. Protocol describing the method to create, copy and modify an electroporation chamber. Click here for file

Additional file 4 **Supplementary Figure 3**. Shapes of the electroporation chambers and electrodes used. Click here for file
