# Supplementary material for: Electroporation of cDNA/Morpholinos to targeted areas of embryonic CNS in Xenopus
Source: BMC Dev Biol. 2007 Sep 27;7:107. doi: 10.1186/1471-213X-7-107 (PMC2147031; doi:10.1186/1471-213X-7-107)
Supplement: Additional file 1 — Supplementary Figure 1. Distribution of early GFP-expressing cells in the brain and eye and stage dependency of eye-targeted electroporation. [file 1471-213X-7-107-S1.pdf]

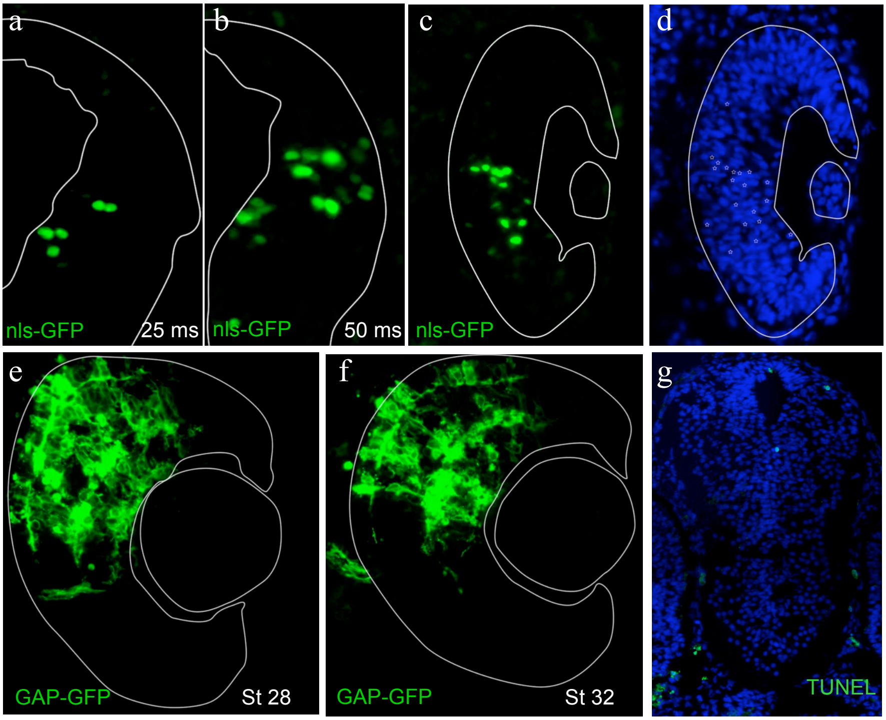

Supplementary Figure 1: Distribution of early GFP-expressing cells in the brain and eye and stage dependency of eye-targeted electroporation

Following intraventricular injection/electroporation at stage 28, the number of nls-GFP positive cells after 6 h doubled when 50 ms pulses (b) were used instead of 25 ms ones (a). More cells were found in the superficial part of the brain in the 50 ms condition (a-b). 6 h post-electroporation, the first nls-GFP cells could be detected in the retina. Some of them were located in the basal half of the central retina, where the early-born RGCs first start to differentiate (c). Eye microanatomy appeared normal at this time point (d). High electroporation efficiency and broad distribution of transfected cells could be achieved within the retinas of embryos electroporated at stage 28 (e) and 32 (f). In both conditions, RGC axons could be observed (arrow heads). g: TUNEL staining of an embryo having received a 18V/25ms/1s/8x pulses stimulation. As in the control condition, only a few TUNEL positive cells (green) were present in the brain. Consistent with the normal pattern of cell death during *Xenopus* development, an accumulation of dying cells was often observed in the optic stalk [1, 2]. Scale bars: 100 µm in g; 50 µm in a and c; 25 µm in e.

1. Hensey C, Gautier J: **Programmed cell death during Xenopus development: a spatio-temporal analysis.** *Dev Biol* 1998, **203:**36-48.
2. Johnston J, Chan R, Calderon-Segura M, McFarlane S, Browder LW: **The roles of Bcl-xL in modulating apoptosis during development of Xenopus laevis.** *BMC Dev Biol* 2005, **5:**20.
