# Supplementary material for: Electroporation of cDNA/Morpholinos to targeted areas of embryonic CNS in Xenopus
Source: BMC Dev Biol. 2007 Sep 27;7:107. doi: 10.1186/1471-213X-7-107 (PMC2147031; doi:10.1186/1471-213X-7-107)
Supplement: Additional file 2 — Supplementary Figure 2. Potential applications of electroporation to other animal models and projection systems. [file 1471-213X-7-107-S2.pdf]

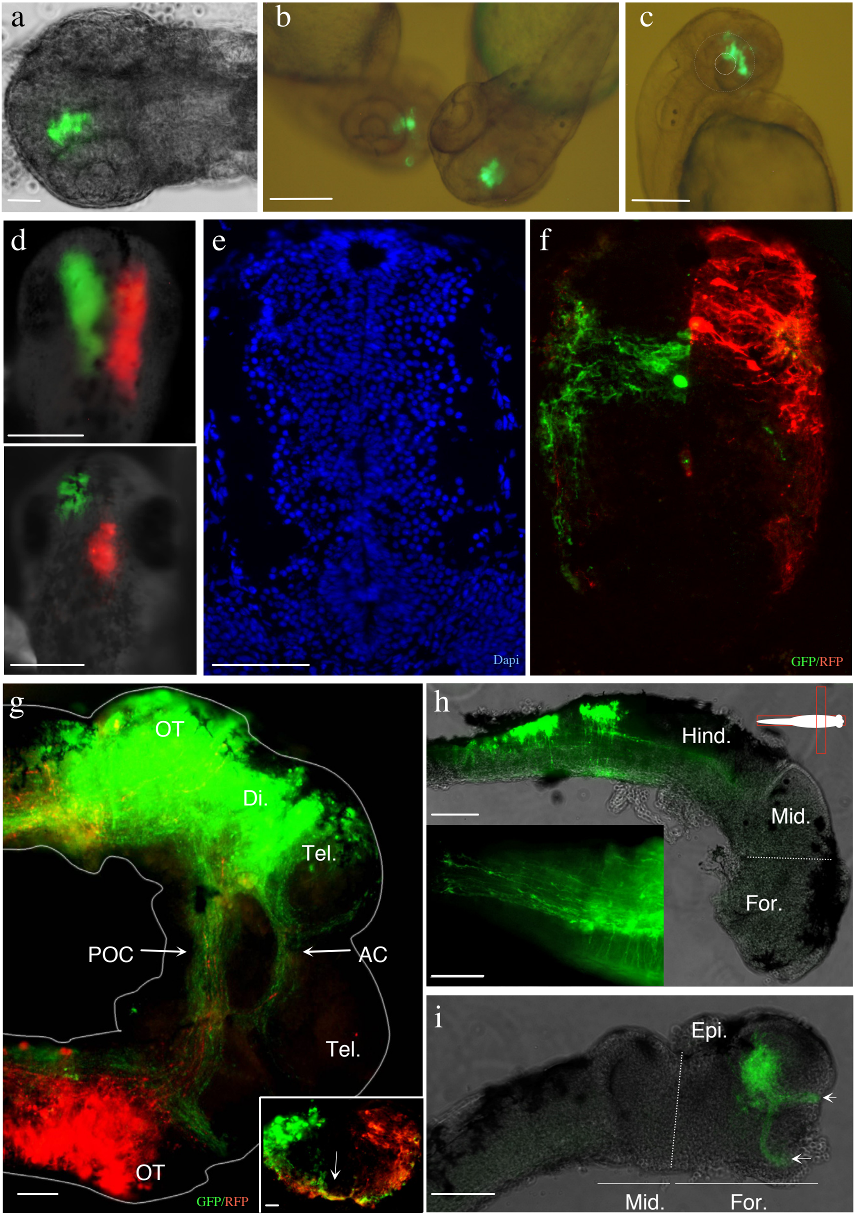
a
b
c
d
e
Dapi
f
GFP/RFP
g
OT
Di.
Tel.
POC
AC
Tel.
OT
GFP/RFP
h
Hind.
Mid.
For.
i
Epi.
Mid.
For.

Supplementary Figure 2: Potential applications of electroporation to other animal models and projection systems

a-c: Zebrafish embryos electroprorated with GFP cDNA using similar chambers (see Supplementary Fig 3a). a: Dorsal view of a brain from an embryo electroporated 30 hpf with GAP-GFP. Live embryos under a fluorescence strereomicroscope exhibiting brain (b) or eye (c) electroporation. d: Dorsal view of double electroporated *Xenopus* embryos. RFP encoding plasmid was electroporated into the left side of the brain and GFP into the right. Both extensive (top) and targeted (bottom) electroporations can be performed. Brain histology (e) and morphology of the transfected cells (f) appears normal on frontal sections of double electroporated embryos. Axons forming the different commissures (arrows), and intermingling with the contralateral brain, can be observed in open-book preparations (g). Commissural axons can also be examined on sections (insert, hindbrain). A dissected brain preparation showing that the caudal hindbrain can be selectively transfected by adjusting the transverse channel location (h). Telencephalon targeted electroporation allows monitoring of the anterior commissural projection (i). AC anterior commissure; Di., diencephalons; Epi. epiphysis; For., forebrain, Mid., midbrain; POC, post-optic commissure; OT optic tectum, Tel., telencephalon. Scale bars: 400 µm in b; 250 µm in b and c; 200 µm in h; 100 µm in a, e, g and i; 50 µm in h insert; 25 µm in g insert.
