# Supplementary material for: Electroporation of cDNA/Morpholinos to targeted areas of embryonic CNS in Xenopus
Source: BMC Dev Biol. 2007 Sep 27;7:107. doi: 10.1186/1471-213X-7-107 (PMC2147031; doi:10.1186/1471-213X-7-107)
Supplement: Additional file 3 — Supplementary Methods. Protocol describing the method to create, copy and modify an electroporation chamber. [file 1471-213X-7-107-S3.doc]

**Carving an electroporation chamber:**

1 volume of curing agent was mixed thoroughly with 10 volumes of base reagent (Sylgard184, Dow corning, USA). The mixture was poured into 3.5 cm dishes and left to polymerize at RT for 24 h (polymerization can be sped up by incubating the dishes in a 50°C incubator). Taking into account the embryo dimensions and the desired type of targeting, a rough blueprint of the chamber was drawn on a Milimetric grid. This blueprint was place under the silicon filled dish to demarcate the channel edges that were to be sculpted with a scalpel blade (15A, Swann-Morton, UK). The channels were progressively carved to the desired depth using a 27G needle (Terumo) and fine forceps. Initial scalpel cuts were also used to give shape to the chamber in the Z-dimension. All the carving was done under the microscope and regular attempts were made during this process to place an embryo into the chamber, so that, regions of the channels that needed further adjustments could be identified. Once the embryos fit properly, test injections were performed to check whether the embryos could be maintained in the correct position. Test electroporations were also carried out to select for chambers that enabled good electroporation without inducing macroscopic damages.

**Making negative imprints, copies and variants of existing chambers**

1 volume of hardener was added to 2 volumes of resin A (Crystal resin, Gedeo, France) in a 14 ml tube (352059, Falcon) and gently mixed by inversion until the solution appeared homogenous (avoiding the creating bubbles as much as possible). Selected chambers were cleaned with water, dried and filled with the resin mix. In some cases, mineral oil was used to lubricate the chambers. Excess oil was carefully removed under the microscope with small pieces of tissue. The resin was pipetted sideways into the chamber with a P200 so that no air gets trapped in. A fine glass tool was used to mix the solution within the chamber and remove the small bubbles. The size of the resin drop was gradually increased so that the solution would spread and create flat circular areas above the chamber channels in the negative imprints (copies of the chambers would be surrounded by a large flat area). This reduces the trapping of medium on any irregular surfaces surrounding the channels, which might change the volume of the electroporation buffer during an experiment. Since the crystal resin moulds sink into Sylgard, the negative imprints of chambers were glued to the bottoms of 3.5 cm Petri dishes. In addition, having the mould at the bottom of a dish prevents the accumulation of small bubbles around it, since air bubbles sticking onto the resin during casting can be removed with a glass tool. The Sylgard level should be high enough so that the chamber does not deform when the embryo or electrodes are being manipulated (approximately 0.8 cm). Sylgard should be left to polymerize at room temperature to reduce the number of trapped bubbles and to preserve the integrity of the resin mould.

Resin moulds can be modified with scalpel blades or modeling tools such as mini-sanders and drills (electric mini-tools, Rotacraft). Modifications to the resin mould are particularly useful for reducing channel depths, as it is not easy to judge whether the channel depth is correct during carving. This enables rapid generation of variants so that the placement of an embryo could be refined or some imperfection corrected.
