# Supplementary material for: Electroporation of cDNA/Morpholinos to targeted areas of embryonic CNS in Xenopus
Source: BMC Dev Biol. 2007 Sep 27;7:107. doi: 10.1186/1471-213X-7-107 (PMC2147031; doi:10.1186/1471-213X-7-107)
Supplement: Additional file 4 — Supplementary Figure 3. Shapes of the electroporation chambers and electrodes used. [file 1471-213X-7-107-S4.pdf]

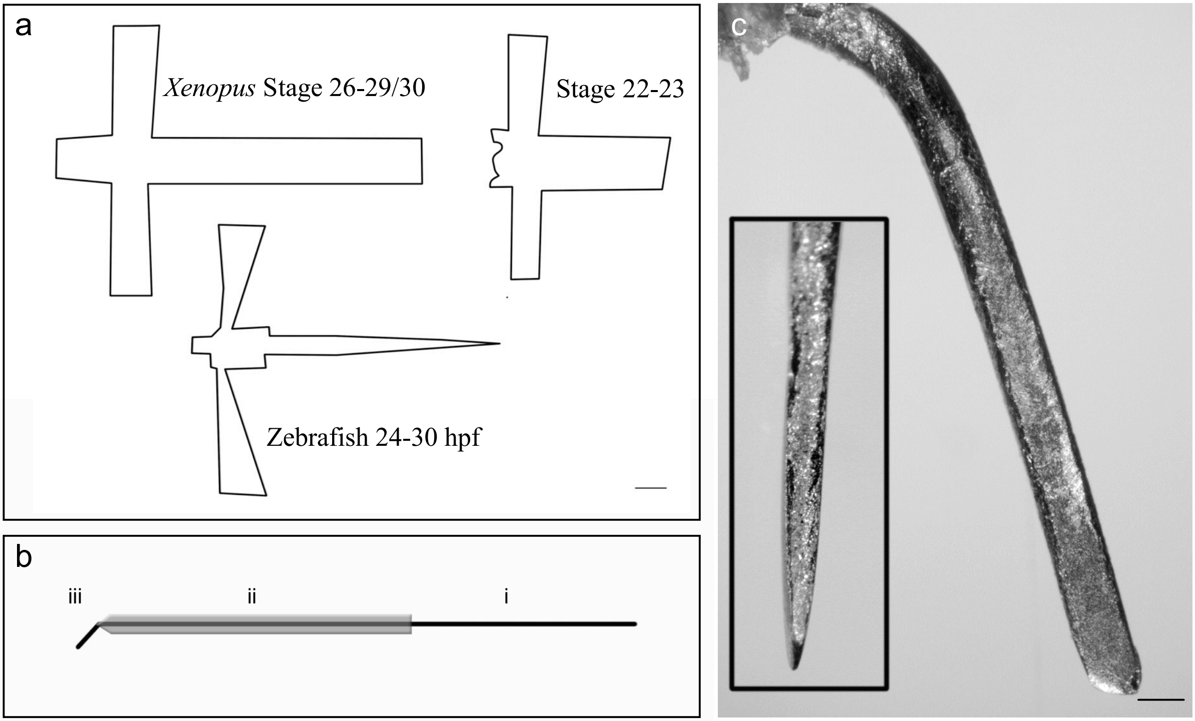

Supplementary Figure 3: Shape of electroporation chambers and electrodes
a: Outlines of the electroporation chambers used to electroporate stage 26-29/30 (top left), stage 22-23 *Xenopus* (top rigth) and 24-30 hpf Zebrafish embryos (bottom).The square region in the bottom chamber was designed to fit the yolk. The outlines were drawn based on photographs of fast green filled chambers. b and c: A schematic diagram of the electrodes used (b) and front and side-view (insert) photographs of the electrode tip (c). The electrode was made from a 0.5 mm platinum wire, and can be divided into three segments. The first segment (i) is for insertion into the electrode holders (Ajustatrode, Intracel). The insulated segment (ii) provides a good working distance. For the first pair of electrodes designed, this region was insulated with nail polish. However, nail polish breaks with time and cannot be used to coat the wires evenly making it difficult to adjust the position and orientation of the electrodes. Therefore, for the second pair of electrodes, plastic sheaths (ID: 0.5 mm), surrounded by metallic sheaths (ID: 1 mm) sealed at both ends with nail polish, were used for insulation. The metallic sheath was added to prevent the platinum wire from bending. Electrode tips (iii) were flattened and polished using an electrical sander and sharpening stones. The 45° bend facilitates placement of the electrodes within the transverse channel. Scale bars 0.5 mm.
